# Urinary Uromodulin Excretion Predicts Progression of Chronic Kidney Disease Resulting from IgA Nephropathy

**DOI:** 10.1371/journal.pone.0071023

**Published:** 2013-08-22

**Authors:** Jingjing Zhou, Yuqing Chen, Ying Liu, Sufang Shi, Suxia Wang, Xueying Li, Hong Zhang, Haiyan Wang

**Affiliations:** 1 Renal Division, Department of Medicine, Peking University First Hospital, Beijing, China; 2 Laboratory of Electron Microscopy, Peking University First Hospital, Peking University First Hospital, Beijing, China; 3 Department of Statistics, Peking University First Hospital, Beijing, China; University of Washington, United States of America

## Abstract

**Background:**

Uromodulin, or Tamm-Horsfall protein, is the most abundant urinary protein in healthy individuals. Recent studies have suggested that uromodulin may play a role in chronic kidney diseases. We examined an IgA nephropathy cohort to determine whether uromodulin plays a role in the progression of IgA nephropathy.

**Methods:**

A total of 344 IgA nephropathy patients were involved in this study. Morphological changes were evaluated with the Oxford classification of IgA nephropathy. Enzyme Linked Immunosorbent Assay (ELISA) measured the urinary uromodulin level on the renal biopsy day. Follow up was done regularly on 185 patients. Time-average blood pressure, time-average proteinuria, estimated glomerular filtration rate (eGFR) and eGFR decline rate were caculated. Association between the urinary uromodulin level and the eGFR decline rate was analyzed with SPSS 13.0.

**Results:**

We found that lower baseline urinary uromodulin levels (P = 0.03) and higher time-average proteinuria (P = 0.04) were risk factors for rapid eGFR decline in a follow-up subgroup of the IgA nephropathy cohort. Urinary uromodulin level was correlated with tubulointerstitial lesions (P = 0.016). Patients that had more tubular atrophy/interstitial fibrosis on the surface had lower urinary uromodulin levels (P = 0.02).

**Conclusions:**

Urinary uromodulin level is associated with interstitial fibrosis/tubular atrophy and contributes to eGFR decline in IgA nephropathy.

## Introduction

Uromodulin (Tamm-Horsfall protein; Uniprot P07911), is an 80–90 kDa glycoprotein, exclusively synthesized by the thick ascending limb (TAL) and early distal convoluted tubule (DCT) in the kidney [1], [2]. Uromodulin *gene (UMOD)* mutations have been confirmed in patients with familial juvenile hyperuricemia nephropathy (FJHN, OMIM 162000), medullary cystic kidney disease 2 (MCKD2, OMIM 603860) and glomerulocystic kidney disease (GCKD, OMIM 609886). Hyperuricemia, hypertension, decreased urinary uromodulin levels, tubulointerstitial nephropathy and progressive kidney disease are characteristics of these diseases [3], [4]. Genome-wide association studies have also revealed that common variants in the promoter region of the *UMOD* gene are associated with chronic kidney disease (CKD), glomerular filtration rate (GFR), kidney stone formation and hypertension [5], [6], [7], [8]. In a previous study, *UMOD* ablation mice presented more inflammation and tubular necrosis in the face of ischemia-reperfusion injury, suggesting that uromodulin stabilizes the outer medulla of the kidney [9]. However it is still unclear whether uromodulin influences the progression of chronic kidney disease.

IgA nephropathy (IgAN) is a common glomerulonephritis and is characterized by a wide range of phenotypes and pathological changes, particularly variable tubular atrophy and interstitial fibrosis [10], [11], [12]. We examined an IgAN cohort to explore whether urinary uromodulin levels at baseline are associated with the progression of IgA nephropathy.

## Methods

### Subjects

This study has been approved by Clinical Research Ethics Committee of Peking University First Hospital. All individuals included in this study had signed consent forms so that their information could be stored in the hospital database and used for research.

#### IgA Nephropathy cohort

All subjects were recruited from the IgAN database in the Renal Division at Peking University First Hospital (http://www.renal-online.org). A total of 344 individuals (195 males and 149 females) were included in this study. Urine samples were collected on the day of renal biopsy and kept at −80°C (Table 1). Regular follow-up was done in 185 individuals of the original cohort (N = 344). Baseline characteristics of the follow-up subgroup did not differ either from the original cohort or from the other 159 individuals that had no follow-up data (Table 1).

**Table 1:** Characteristics of the IgAN cohort in this study.

	N1 = 344	N2 = 185	N3 = 159	P1	P2
				(N1&N2)	(N2&N3)
**Baseline Data**					
Age (years)	33.3±0.7	32.7±0.9	35.6±1.1	0.617	0.045
Male/Female (N)	195/149	112/73	95/64	0.392	0.881
SBP (mmHg)	118.9±0.9	118.3±1.0	119.9±1.4	0.636	0.349
DBP (mmHg)	75.1±0.8	75.0±1.3	75.4±1.4	0.936	0.826
MAP (mmHg)	93.2±0.8	93.1±0.9	91.6±1.0	0.9	0.52
UP (g/24h)	1.48 (0.82∼2.87)	1.45(0.78∼2.74)	1.69(0.78∼2.82)	0.641	0.782
PCr (μmol/L)	105.8±3.9	100.4±2.6	107.5±5.1	0.249	0.2
TC (mmol/L)	4.55±0.12	5.0±0.1	4.6±0.2	0.012	0.05
TG (mmol/L)	1.6(0.9∼2.4)	1.7(1.1∼2.7)	1.5(0.9∼2.5)	0.037	0.055
eGFR (ml/min/1.73m2)	83.7±1.6	84.4±2.3	80.5±2.3	0.799	0.236
UUMOD (μg/ml)	2.6(1.6∼4.2)	2.7(1.7∼4.3)	2.7(1.6∼3.9)	0.714	0.478
Oxford score					
M 0/1(%)	54.1/45.9	51.6/48.4	56.6/43.4	0.673	0.562
E 0/1(%)	67.7/32.3	62.5/37.5	72.3/27.7	0.44	0.058
S 0/1(%)	52.3/47.7	42.4/57.6	52.2/47.8	0.035	0.078
T 0/1/2 (%)	80/10/10	76.1/12.5/11.4	78.6/12.6/8.8	0.517	0.737
**Follow-up data**					
Time (month)		39.7±1.8			
TA-MAP (mmHg)		89.2±0.6			
TA-proteinuria (g/24h)		0.86(0.51∼1.52)			
eGFR decline rate		−0.144(−0.465∼0.113)			
Renal endpoint (N)		13			
ACEi and/or ARB		100%			
Steroid and/or CTX		42%			

Data are presented as mean ± SEM or median (inter-quartile range [IQR]) for continuous variables and proportions for categorical variables. SBP: Systolic blood pressure; DBP: Diastolic blood pressure; MAP: Mean arterial blood pressure; UP: Urinary protein; PCr: Plasma creatinine; TC: Total cholesterol; TG: Triglyceride; UUMOD: Urinary uromodulin; TA-MAP: Time-average blood pressure; TA-proteinuria: Time-average proteinuria. ESKD: End stage kidney disease. ACEi: Angiotensin I Converting Enzyme Inhibitor. ARB: Angiotensin receptor blocker. CTX: Cyclophosphamide. 100% of follow-up patients (N = 185) accepted ACEi and/or ARB therapy. 42% of patients accepted steroid and/or cyclophosphamide. eGFR was calculated with the Chronic Kidney Disease Epidemiology Collaboration (CKD-EPI) equation [15]. eGFR decline rate: was obtained by fitting a straight line through the calculated eGFR using linear regression and the principal of least squares for every patient [13]. N1: the whole cohort; N2: the subgroup with follow-up; N3: the subgroup without follow-up data.

Altogether, 185 individuals were followed up for more than 3 years (39.7+/−1.8 months). Blood pressure, plasma creatinine, urinary proteinuria and other physical/biochemical characteristics of these individuals were collected every 3 months during the follow-up period. We calculated time average mean blood pressure (TA-MAP) and time average 24-hour proteinuria (TA-proteinuria), according to a previous report [13]. Estimated GFR (eGFR) was calculated with the equation developed from data based on Chinese patients with CKD [14], as well as the Chronic Kidney Disease Epidemiology Collaboration (CKD-EPI) equation [15]. These two sets of eGFR caculations were highly correlated, thus we used the CKD-EPI eGFR for further analyses. During follow-up, plasma creatinine was measured every 3 months in each person, and the renal function decline during follow-up was represented by slope of eGFR, which was obtained by fitting a straight line through the calculated eGFRs using linear regression and the principal of least squares [13], so that each person has his/her own independent eGFR decline rate. All of 185 follow-up patients took angiotensin I converting enzyme inhibitor or/and angiotensin receptor blocker (ACEi or/and ARB), and 42% patients took steroids and/or cyclophosphamide (CTX) (Table 1). Characteristics of the two subgroups are presented in table 2.

**Table 2:** Characteristics of 185 IgAN patients with follow up data stratified by steroids/CTX.

	With steroid/CTX	Without Steroid/CTX	P
	N = 78	N = 107	
**Baseline data**			
Age (years)	31.3±1.4	33.7±1.2	0.185
Male/Female (N)	44/34	68/39	0.362
MAP (mmHg)	92.6±1.3	93.5±1.1	0.588
UP (g/24h)	2.0 (0.97∼4.18)	1.17(0.68∼2.11)	0.007
eGFR(ml/min/1.73m^2^)	83.9±3.6	84.7±3.0	0.877
UUMOD (μg/ml)	2.5(1.6∼4.2)	2.8(1.8∼4.4)	0.318
Oxford score			
M 0/1(%)	35.9/64.1	63.6/36.4	0.001
E 0/1(%)	52.6/47.4	70.1/29.9	0.021
S 0/1(%)	33.3/66.7	49.5/50.5	0.02
T 0/1/2 (%)	67.9/16.7/15.4	82.2.1/9.3/8.5	0.079
**Follow-up data**			
Time (month)	43.4±2.7	37.1±2.3	0.073
TA-MAP (mmHg)	89.3±0.8	89.1±0.9	0.862
TA-proteinuria (g/24h)	1.12(0.62∼1.78)	0.77(0.47∼1.22)	0.008
EGFR decline rate	−0.246(−0.559∼0.101)	−0.054(−0.363∼0.178)	0.051
ACEi and/or ARB	100%	100%	

Data are presented as mean ± SEM or median (inter-quartile range [IQR]) for continuous variables and proportions for categorical variables. MAP: Mean arterial blood pressure; UP: Urinary protein; UUMOD: Urinary uromodulin; TA-MAP: Time-average blood pressure; TA-proteinuria: Time-average proteinuria. ACEi: Angiotensin I Converting Enzyme Inhibitor. ARB: Angiotensin receptor blocker. CTX: Cyclophosphamide. eGFR was calculated with the Chronic Kidney Disease Epidemiology Collaboration (CKD-EPI) equation [15]. eGFR decline rate: was obtained by fitting a straight line through the calculated eGFR using linear regression and the principal of least squares for every patient [13].

### Evaluation of renal pathological changes

The pathological changes were re-evaluated with the Oxford classification system of IgAN. All available microscopic slides were reviewed for each case. Four histological parameters were evaluated as follows: 1) the mesangial hypercellularity score (M0≤0.5/M1>0.5, if more than half of the glomeruli had more than three cells in a mesangial area, this was categorized as M1, otherwise as M0); 2) endocapillary hypercellularity (hypercellularity due to an increased number of cells within the glomerular capillary lumina causing narrowing of the lumina, E0-absent/E1-present); 3) segmental glomerulosclerosis (any amount of the tuft involved in sclerosis, but not involving the whole tuft or the presence of an adhesion, S0-absent/S1-present); 4) Interstitial fibrosis/tubular atrophy (percentage of cortical area covered by the tubular atrophy or interstitial fibrosis, whichever is greater, T0: 0–25%/T1: 26–50%/T2: >50%) [16], [17] (Tables 1& 2).

### Biochemical assays

Enzyme Linked Immunosorbent Assay (ELISA) measured urinary uromodulin levels according to Lau's protocol with minor modifications [18]. Microtiter plates were coated with rabbit anti-human uromodulin glycoprotein polyclonal antibody (Biomedical Technologies Inc, BT-590, Stoughton, MA, USA). Urine samples from IgA nephropathy patients and uromodulin standards (MY Biosource,Uromodulin Human Native protein,MBS364097, SanDiego, CA, USA) were added to wells in duplicate. The mouse anti-human uromodulin protein Monoclonal antibody was added as a second antibody (Cedarlane,CL1032 AP, Burlington, NC, USA). Goat anti-mouse IgG horseradish peroxidase was added as a third antibody. Urinary uromodulin level was determined by referring to a standard curve. Urinary creatinine was measured on a Hitachi 7170 autoanalyzer (Hitachi, Tokyo, Japan). Both urinary uromodulin concentration and urinary uromodulin over creatinine ratio were calculated, and were found to correlated very well (R = 0.7, P<0.001), thus urinary uromodulin concentration was used to do the analyses.

### Statistical analyses

The Statistical Package for the Social Sciences (SPSS v13.0; Chicago, IL, USA) was used. Baseline characteristics were reported as mean ± SEM or median (inter-quartile range [IQR]) for continuous variables and proportions for categorical variables. The non-parametric Mann–Whitney test and the independent samples t test were used to test for differences between groups.

The primary endpoint was defined as a 50% eGFR decline or end stage kidney disease (ESKD). Secondary endpoint was renal function decline (the eGFR slope). During follow-up, plasma creatinine was measured every 3 months for each person, so that each person has his/her own eGFR slope.

Cox regression analysis was used to test whether urinary uromodulin level was a risk factor for ESKD or for a 50% decline of eGFR. Linear regression analyses were used to test whether urinary uromodulin level predicted renal function decline in IgAN. Renal function decline, defined as the eGFR slope, was used as a dependent variable. Physical/biochemical factors, such as age, gender, baseline-eGFR, baseline-proteinuria, baseline-MAP, TA-proteinuria, TA-MAP, baseline urinary uromodulin and pathological items (M/E/S/T) and type of treatments (with steroid/CTX & without steroid/CTX) were analyzed with single factor followed by multiple factors linear regression. Two models were set for linear regression. Uromodulin level was analyzed as a continuous variable in model I and as a categorical variable (low vs. high) in model II.

Univariate analysis was performed to detect any association of tubular atrophy/interstitial fibrosis and gender with urinary uromodulin levels.

## Results

### Urinary uromodulin excretion associated with progression of IgA nephropathy

In the linear regression analyses, urinary uromodulin level was analyzed first as a continuous variable in model I and then as a categorical variable in model II. In model I, we found that urinary uromodulin levels were associated with eGFR decline in the follow-up subgroup of the IgAN cohort (P = 0.04, Table 3). Individuals with lower urinary uromodulin levels had faster eGFR decline during follow-up (Table 3). In model II, we assigned individuals into two groups (low vs. high) according to the median value of the urinary uromodulin levels. Again, model II showed that individuals in the low uromodulin group had a faster eGFR decline compared with individuals in the high uromodulin group (P = 0.03, Table 3). In both models TA-proteinuria was a predictor of eGFR decline, and those with higher TA-proteinuria had a faster decrease in eGFR. Other physical/biochemical factors, such as age, gender, baseline-eGFR, baseline-proteinuria, baseline-MAP, TA-MAP, pathological items and type of treatments were analyzed with single factor and then multiple factors linear regression and were not found to be associated with eGFR decline in either of these two models.

**Table 3:** Urinary uromodulin excretion contributed to renal function decline in the IgAN cohort (N = 185).

	Standarized β	95%CI	P
**Model 1**			
TA-UP	−0.162	−0.551∼−0.092	0.03
UUMOD (ug/ml)	0.159	4.8*10^−6^∼1.6*10^−4^	0.04
**Model 2**			
TA-UP	−0.159	−0.459∼−0.015	0.04
UUMOD (Low vs. high)	−0.162	−0.840∼−0.033	0.03

Analyses were performed using linear regression. Renal function decline was chosen as a dependent variable. Uromodulin was analyzed as a continuous variable in model I, and as a categorical variable in model II. In each analysis, physical and biochemical traits including age, gender, baseline-eGFR, baseline-MAP, baseline urinary protein, baseline urinary uromodulin, pathological changes (scored by Oxford system as M E S T ), time average MAP, time average urinary protein and type of treatment (with or without steroid/CTX) were first analyzed by single factor analysis. Baseline-eGFR, baseline urinary uromodulin, baseline urinary protein and TA-UP were associated with eGFR decline. Thus these four factors were included in the multiple factor analysis. The result from multiple factor analysis is presented. UUMOD: Urinary uromodulin; TA-UP: Time-average proteinuria.

A total of 13 cases in the follow-up subgroup reached the primary endpoint (defined as a 50% eGFR decline or ESKD). We performed single factor as well as multiple factors Cox regression and found that urinary uromodulin level was not a risk factor, but that TA-proteinuria did appear to be a risk factor for the renal endpoint in IgAN (HR = 3.118, 95%C.I. 1.938–5.017, P<0.001).

### Clinical and pathological factors contributed to urinary uromodulin excretion

Since urinary uromodulin levels appeared to be associated with IgAN progression, we further explored which clinical/pathological factors contributed to urinary uromodulin levels in IgAN.

**Figure 1:**
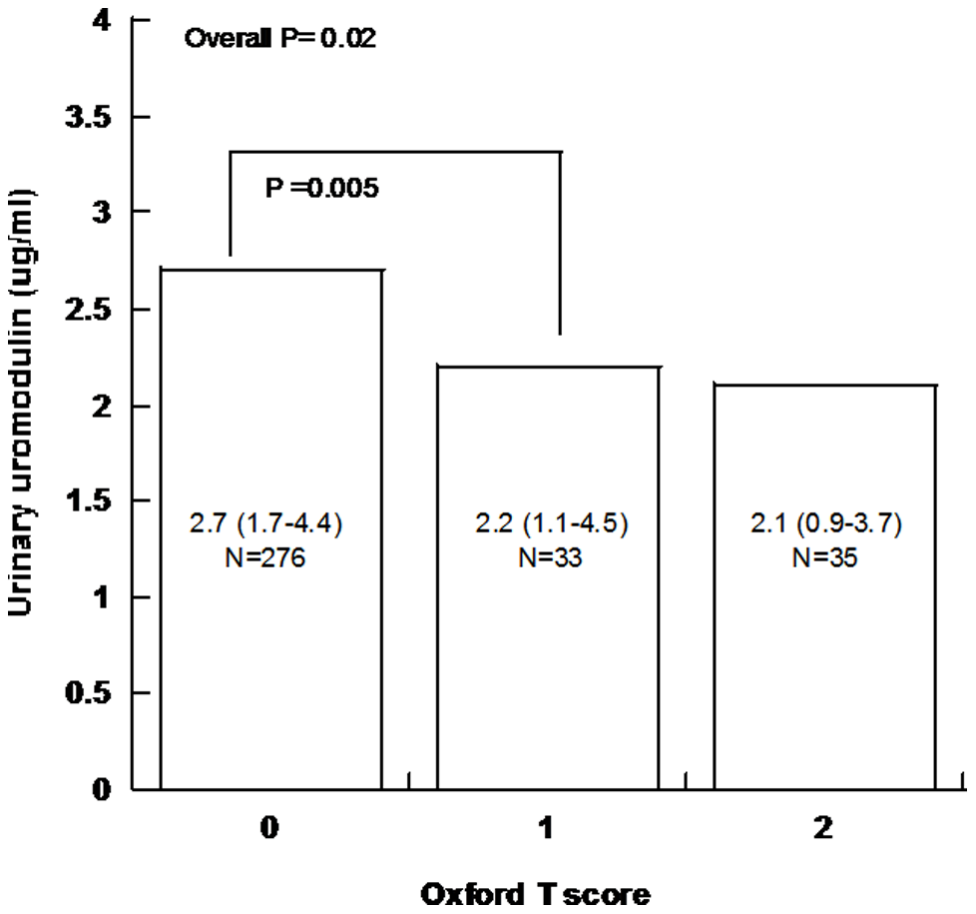
Urinary uromodulin levels association with interstitial fibrosis/tubular atrophy. Median urinary uromodulin level is presented (inter-quartile range [IQR]). All individuals were divided into three groups according to their Oxford interstitial fibrosis/tubular atrophy score. 0: 0–25% 1: 26%–50% 2: >50%. Non-parametric Mann–Whitney test was used to test the difference in urinary uromodulin levels among the three groups.

**Figure 2:**
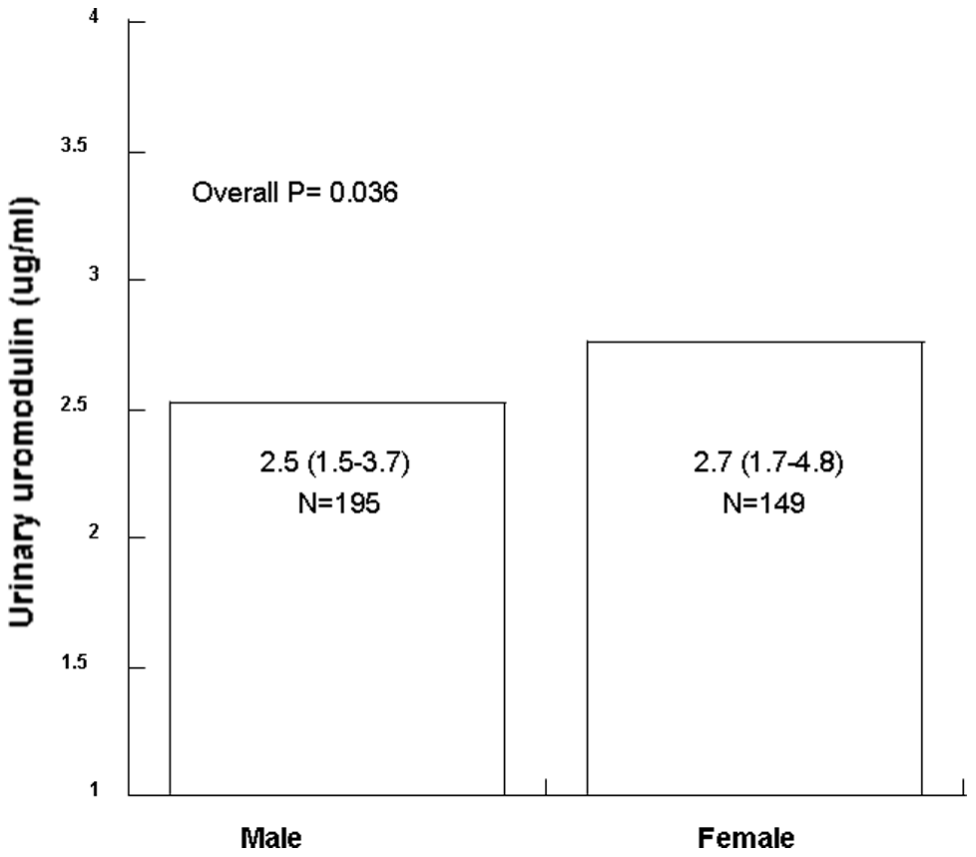
Urinary uromoudulin levels association with gender. Median urinary uromodulin level is presented (inter-quartile range [IQR]). Non-parametric Mann–Whitney test was used to test the difference in urinary uromodulin levels between the two groups.

In the linear regression analysis, urinary uromodulin levels were selected as the dependent variable, and physical/biochemical factors such as age, gender, baseline-eGFR, baseline-proteinuria, SBP, DBP and pathological items (M/E/S/T) were analyzed with single factor and then multiple factors models. Using the multiple factors model, we found that gender (P = 0.032) and tubular atrophy/interstitial fibrosis (P = 0.016) were associated with urinary uromodulin levels (Table 4). Patients with more tubular atrophy and interstitial fibrosis had lower concentrations of urinary uromodulin (P = 0.016, Table 4 & Figure 1), and the urinary uromodulin level in females was higher than that in males (P = 0.036, Table 4 & Figure 2). Other clinical/pathological parameters, such as baseline eGFR, proteinuria, blood pressure, mesangial hypercellularity, endocapillary hypercellularity and segmental glomerulosclerosis, did not correlate with urinary uromodulin levels.

**Table 4:** Clinical and pathological factors contributed to urinary uromodulin levels.

	Standarized β	95%CI	P
**Gender**	0.117	54.4∼1161	0.032
**T**	−0.132	−948.796∼−100.179	0.016

Analyses were performed with linear regression. Physical and biochemical traits, including age, gender, baseline eGFR, baseline blood pressure, baseline urinary protein and pathological changes (scored by Oxford system as M E S T ), were analyzed by single factor analysis first, then followed by multiple factor analysis. The result from multiple factor analysis is presented. T: tubulointerstitial score.

## Discussion

Uromodulin has been known for more than 50 years [19]. Since it's discovery, a great deal of research has been done illustrating novel roles of this protein. Previous studies have indicated that *UMOD* mutations contribute to FJHN/MCKD2 [4] and that promoter variants of the *UMOD* gene are associated with eGFR, blood pressure, plasma uric acid level, and incidence of chronic kidney disease [5], [6], [7], [8]. However it is still unclear whether uromodulin influences the progression of chronic kidney disease. IgA nephropathy is the most common primary glomerulonephritis and is characterized by wide range of phenotypes and variable pathological changes, especially tubular and interstitial lesions [10], [11], [12], thus an IgAN cohort was thought to be a more suitable model for this study, compared to cohorts with other glomerular diseases.

According to previous reports, baseline eGFR, TA-proteinuria and TA-MAP are strong predictors of IgAN progression [13]. Here we found that urinary uromodulin levels (P = 0.04) at baseline and TA-proteinuria (P = 0.03) were associated with the renal function decline in the IgAN cohort. It is obvious that treatment with steroid/cyclophosphamide (CTX) was not randomized among these patients and the subgroup treated with steroid/CTX treatment had higher baseline proteinuria compared with the group that did not receive steroid/CTX treatment (P = 0.007, Table 2). The time average proteinuria was still higher in the subgroup treated with steroid/CTX compared with the other group (P = 0.008, Table 2), although steroid/CTX was added after ACEi/ARB. Thus the types of treatments were also added as independent variables in the regression model, but did not show significant associations with renal function decline.

We also found that urinary uromodulin level was associated with tubulointerstitial lesions. Patients with more surface tubular atrophy/interstitial fibrosis excreted less uromodulin into the urine (P = 0.016, Table 4). Tubular cells in the corticomedullary junction exclusively express uromodulin and the corticomedullary junction can be easily injured. Thus uromodulin synthesis and excretion will be influenced when tubular atrophy/interstitial fibrosis occurs. In other renal disease, such as autosomal polycystic kidney disease, glomerular nephritis and diabetic nephropathy, urinary uromodulin excretion is obviously decreased [20], [21], [22], [23]. Here we further found that tubular atrophy/ interstitial fibrosis to be associated with urinary uromodulin levels in IgA nephropathy. Patients with more tubular atrophy and interstitial fibrosis had lower urinary uromodulin levels. Uromodulin excretion also seems to increase gradually from birth to adulthood, becomes stable [24], [25], [26], [27] and then begins to decline after 60 years of age [28], [29]. We did not indentify an association between age and urinary uromodulin levels, probably due to narrow range of ages in this cohort. eGFR was not a correlated factor either, but severity of tubular atrophy and interstitial fibrosis did correlate with urinary uromodulin excretion in this cohort.

Uromodulin may be only a marker of tubular damage, or it could also be a factor involved in the pathogenic process of renal disease. Evidence from studies with human and mice [9], [21], [30], [31] suggest that the role of uromodulin is multi-faceted. One study found that elevated urinary uromodulin level was a risk factor for future incidence of chronic kidney disease [6]. However, in diabetes type I, it was shown that decreased uromodulin excretion was a predictive factor for renal failure and cardiovascular disease in adults [21]. A study with *UMOD* knockout mice indicated that uromodulin stabilized the outer medulla of the kidney in face of injury by decreasing inflammation [9].

Although both pathologic changes and urinary uromodulin levels were included in the models, only urinary uromodulin levels were associated with eGFR decline. This result indicates that urinary uromodulin is an independent clinical factor associated with progression of IgA nephropathy. Uromodulin might play a two-faceted role in chronic kidney disease: the damage of renal tubules may result in decreased synthesis of uromodulin and uromodulin itself also involved in the pathogenesis of kidney disease progression.

As a pilot study with limited number of subjects, followed-up time and events, the power for detecting an association between urinary uromodulin and endpoints, especially in slowly progressing disease was low. We defined composite primary endpoints (ESKD or 50% eGFR decline), only TA-proteinuria, and not uromodulin, was found to have an effect on the endpoints in this study. However, we did find that both time average proteinuria and uromodulin were associated with eGFR decline, which is a commonly used endpoint in kidney disease. More subjects and a longer follow-up time are needed in future studies.

In summary, we found that urinary uromodulin levels are associated with interstitial fibrosis/ tubular atrophy and contribute to eGFR decline in IgAN, implicating a protective role of uromodulin in kidney disease progression. Uromodulin excretion decreased with tubulointerstitial lesions, leading to weakened protection and disease progression.
